# Gender-specific association of the rs6499640 polymorphism in the *FTO* gene with plasma lipid levels in Chinese children

**DOI:** 10.1590/1678-4685-GMB-2017-0107

**Published:** 2018-06-04

**Authors:** Liwang Gao, Lijun Wu, Meixian Zhang, Xiaoyuan Zhao, Hong Cheng, Jie Mi

**Affiliations:** 1 Capital Institute of Pediatrics Capital Institute of Pediatrics Department of Epidemiology Beijing China Department of Epidemiology, Capital Institute of Pediatrics, Beijing, China

**Keywords:** children, FTO, lipid levels

## Abstract

The fat mass- and obesity-associated gene (*FTO*) is significantly associated with obesity, but the associations of *FTO* with obesity-related traits are not fully described. We aimed to investigate the association of the *FTO* single nucleotide polymorphism (SNP) rs6499640 with lipid levels in Chinese children. A total of 3503 children aged 6-18 years were included in the present study. Lipid levels were analyzed and the SNP rs6499640 was genotyped using the TaqMan Allelic Discrimination Assay. Statistically significant associations were found between rs6499640 and low-density lipoprotein cholesterol (LDL-C) (*p* = 0.008), total cholesterol (TC) (*p* = 0.005), and triglycerides (TG) (*p* < 0.001) in girls under a dominant model adjusted for age and BMI. No statistical significance was found between the SNP and lipid levels in boys. We demonstrated for the first time that the SNP rs6499640 in *FTO* is associated with LDL-C, TC, and TG in Chinese girls. Our study identified a new risk locus for lipid levels in children.

## Introduction

In recent years, the prevalence of dyslipidemia and obesity in children and adolescents has increased dramatically and these two diseases have been major challenges to public health (Keith *et al.*, 2006; Miller *et al.*, 2011; Stone *et al.*, 2014; Jacobson *et al.*, 2015). Childhood high lipid levels and obesity strongly predispose to adult cardiovascular disease (CVD) and obesity (Tounian *et al.*, 2001; Simmonds *et al.*, 2015).

Lipid levels and obesity are complex conditions influenced by genetic and environmental factors (Kathiresan *et al.*, 2008). Family and twin studies have indicated that more than 50% of changes in lipid levels is ascribed to genetic factors (Kort *et al.*, 2000). The fat mass- and obesity-associated gene (*FTO*) was the first gene associated to obesity in genome-wide association studies (GWAS) (Loos and Bouchard, 2008). Thereafter, many of the single nucleotide polymorphisms (SNPs) of *FTO* have been confirmed to be associated with lipid levels (Scuteri *et al.*, 2007; Wang *et al.*, 2012). Our previous study suggested that the *FTO* SNP rs6499640 was associated with obesity in Chinese children (Wu L *et al.*, 2010). However, the associations of this SNP with lipid levels are not described.

As obesity is closely related to high lipid levels, it is possible that both diseases share a common genetic background. Evidence from several studies indicates that the association between *FTO* variants and lipid levels may be BMI-dependent and may differ by gender (Kring *et al.*, 2008; Fang *et al.*, 2010; Elouej *et al.*, 2016), but different results have been found in other studies (Muñoz-Yáñez *et al.*, 2016; Qureshi *et al*., 2017).

In the present study, we evaluated the associations between *FTO* rs6499640 and lipid levels and examined gender-specific associations in Chinese children.

## Materials and Methods

### Subjects

Subjects were recruited from the Beijing Child and Adolescent Metabolic Syndrome (BCAMS) study (Mi *et al.*, 2006), which was a cross-sectional population-based study that included a questionnaire, medical examination, anthropometric measurement and finger capillary blood test in a representative sample of Beijing school-age children (n=19,593, aged 6-18 years, 50% boys) in 2004. From the BCAMS study, a subgroup of 1229 obese children and 2274 non-obese children were selected. The BCAMS study was approved by the Ethics Committee at the Capital Institute of Pediatrics in Beijing. Written informed consent was signed by participants’ parents or guardians.

### Anthropometric measurements and biochemical analyses

Height and weight were measured according to our standard protocol (Mi *et al.*, 2006). The lipid levels were assessed by enzymatic methods for total cholesterol (TC) and triglyceride (TG), and clearance methods for high-density lipoprotein cholesterol (HDL-C) and low-density lipoprotein cholesterol (LDL-C), using the Hitachi 7060C Automatic Biochemistry Analysis System.

### Definition of obesity and dyslipidemia

Obesity was defined by using the Chinese age- and sex-specific body mass index (BMI) cutoffs (Supplementary Table S1), recommended by the Working Group on Obesity in China (Ji and Working Group on Obesity in China, 2005). Dyslipidemia was diagnosed if the child fell in at least one of the following four categories: 1) high TC: TC ≥ 5.18 mmol/L; 2) high TG: TG ≥ 1.70 mmol/L; 3) high LDL-C: LDL-C ≥ 3.37 mmol/L; 4) low HDL-C: HDL-C ≤ 1.04 mmol/L (Editorial Board of Chinese Journal of Pediatrics *et al.*, 2009).

### Genotyping

Genomic DNA was isolated from peripheral white blood cells using the salt fractionation method. The SNP rs6499640 was genotyped using the TaqMan Allelic Discrimination Assay with the GeneAmp 7900 Sequence Detection System (Applied Biosystems, USA). Genotyping call rates for SNP was > 98%. In order to validate the accuracy of genotyping, we repeated 70 samples randomly for each SNP and observed 100% concordance between the results of the two tests.

### Statistical analysis

Statistical analyses were performed using the statistical software SPSS, version 19.0 (IBM Corp, USA). Quantitative variables were presented as the mean ± standard deviation (SD) and categorical variables were presented as percentages. Hardy-Weinberg equilibrium was assessed using the chi-square test. The difference of lipid levels (LDL-C, TC, TG, and HDL-C) between AA and GG+GA groups was analyzed by covariance analysis. Adjusted odds ratios (ORs) for high LDL-C, high TC, high TG and low HDL-C were computed by logistic regression with genotype, age, gender, and BMI or obesity status as the independent variables. *p* < 0.05 was used to indicate statistically significant differences. False discovery rate (FDR) approach was used to correct for multiple testing. Power calculation was performed using Quanto software ( http://hydra.usc.edu/gxe/).

## Results

Table 1 shows the demographic and clinical characteristics of the study population. We genotyped the SNP rs6499640 in *FTO* in the cohort, and the genotypes of the SNP (the number of GG, GA, and AA was 2401, 932 and 110, respectively) were in Hardy–Weinberg equilibrium (*p* = 0.095). Comparison of characteristics between boys and girls are shown in Table 1. Girls had higher TC, TG, and LDL-C values than boys (all *p* < 0.05).

**Table 1:** Characteristics of the study population.

Characteristics	All	Boys	Girls	*p* ^a^
N	3503	1781	1722	
Age (years)	12.4 ± 3.1	12.2 ± 3.1	12.6 ± 3.1	< 0.001
BMI (kg/m)	21.9 ± 4.9	22.85.2	21.0 ± 4.5	< 0.001
Weight status (%)				< 0.001
Obese	1229(35.1)	787(44.2)	442(25.7)	
Non-obese	2274(64.9)	994(55.8)	1280(74.3)	
TC (mmol/L)	4.09 ± 0.83	4.07 ± 0.78	4.12 ± 0.86	0.035
TG (mmol/L) ^b^	0.92 ± 0.45	0.91 ± 0.47	0.94 ± 0.43	< 0.001
HDL-C (mmol/L)	1.40 ± 0.32	1.39 ± 0.34	1.42 ± 0.30	0.064
LDL-C (mmol/L)	2.55 ± 0.76	2.53 ± 0.71	2.57 ± 0.80	0.006
high TC (%)	313(8.9)	150(8.4)	163(9.5)	0.288
high TG (%)	340(9.7)	188(10.5)	152(8.8)	0.080
low HDL-C (%)	397(11.3)	227(12.7)	170(9.9)	0.007
high LDL-C (%)	426(12.2)	200(11.2)	226(13.1)	0.092
Dyslipidemia (%)	990(28.3)	526(29.5)	464(26.9)	0.080

Data are presented as mean ± standard deviation or percentages of subjects, as appropriate.

BMI, body mass index. TC, total cholesterol. TG, triglyceride. HDL-C, high-density lipoprotein cholesterol. LDL-C, low-density lipoprotein cholesterol. high TC: TC ≥ 5.18 mmol/L, high TG: TG ≥ 1.70 mmol/L, high LDL-C: LDL-C ≥ 3.37 mmol/L, low HDL-C: HDL-C ≤ 1.04 mmol/L; At least 1 category had to be present for dyslipidemia diagnosis.

There were 8, 6, 6, 9, 11 missing data in TG, TC, HDL, LDL, dyslipidemia respectively.

a Comparison between males and females was preformed using age- and BMI- adjusted regression analysis for continuous variables, χ^2^ tests for categorical variables, and t test to examine difference in age and BMI.

b natural logarithm-transformed.

The difference in LDL-C, TC, TG, and HDL-C with different genotypes of rs6499640 are shown in Table 2. There were significant differences in LDL-C (*p* = 0.002), TC (*p* = 0.001), and TG (0.019) with different genotypes (AA, GG+GA) after adjustment for age and gender. As SNP rs6499640 was significantly associated with BMI, we also adjusted for BMI or obesity status besides age and gender. The SNP rs6499640 was significantly different in LDL-C (*p* = 0.002), TC (*p* = 0.001), and TG (0.007) with different genotypes (AA, GG+GA) under the dominant model after adjusting for age, gender, and BMI. The associations of rs6499640 with high LDL-C, high TC, high TG, low HDL-C, and dyslipidemia were also tested, but no significance was found (Table S2).

**Table 2:** Difference in lipid levels with different genotypes of rs6499640.

Traits	Dominant model	N	$\bar{x}$ ± SD	Model 1		Model 2		Model 3	
				Change to AA	*p*	Change to AA	*p*	Change to AA	*p*
				(95%CI)		(95%CI)		(95%CI)	
LDL-C	AA	110	2.34 ± 0.71					
	GG+GA	3325	2.56 ± 0.76	0.23	0.002	0.235	0.002	0.252	0.002
				(0.085-0.376)		(0.090-0.380)		(0.092-0.411)	
TC	AA	110	3.85 ± 0.76						
	GG+GA	3328	4.10 ± 0.83	0.274	0.001	0.274	0.001	0.303	0.001
				(0.116-0.433)		(0.115-0.432)		(0.130-0.477)	
TG^a^	AA	110	0.84 ± 0.43						
	GG+GA	3328	1.08 ± 0.45	0.103	0.019	0.112	0.007	0.103	0.025
				(0.017-0.189)		(0.030-0.193)		(0.013-0.192)	
HDL-C	AA	110	1.41 ± 0.30						
	GG+GA	3328	1.40 ± 0.32	-0.001	0.987	-0.008	0.767	-0.007	0.813

Data are expressed as the mean ± standard deviation.

Model 1: Adjusted for age and gender. Model 2: Adjusted for model 1 + BMI. Model 3: Adjusted for model 1 + obesity status.

TC, total cholesterol; TG, triglyceride; HDL-C, high-density lipoprotein cholesterol; LDL-C, low-density lipoprotein cholesterol; CI, confidence interval.

a natural logarithm-transformed.

Table 3 shows the differences in LDL-C, TC, TG, and HDL-C levels with different genotypes of rs6499640 under the dominant model by gender. After adjusting for age and BMI, we found significant differences in LDL-C (*p* = 0.008), TC (*p* = 0.005), and TG (*p* < 0.001) with different genotypes (AA, GG+GA) in girls. The above results were not found in boys, and there was no association between rs6499640 and high LDL-C, high TC, high TG, low HDL-C and dyslipidemia in boys and girls (Table S2).

**Table 3:** Difference in lipid levels with different genotypes of rs6499640 by gender.

Gender	Traits	Dominant model	N	$\bar{x}$ ± SD	Model 1			Model 2			Model 3	
					Change to AA	*p*		Change to AA	*p*		Change to AA	*p*
					(95%CI)			(95%CI)			(95%CI)	
Boys												
	LDL-C	AA	64	2.44 ± 0.72								
		GG+GA	1675	2.54 ± 0.71	0.144	0.112		0.149	0.095		0.19	0.072
					(-0.033-0.321)			(-0.026-0.324)			(-0.017-0.397)	
	TC	AA	64	3.91 ± 0.77								
		GG+GA	1677	4.07 ± 0.79	0.184	0.063		0.185	0.061		0.243	0.058
					(-0.010-0.378)			(-0.008-0.379)			(0.014-0.472)	
	TG^a^	GG+GA	1676	0.91 ± 0.47								
		AA	64	0.91 ± 0.51	-0.01	0.867		-0.001	0.980		-0.019	0.768
					(-0.127-0.107)			(-0.108-0.106)			(-0.146-0.108)	
	HDL-C	AA	64	1.37 ± 0.29								
		GG+GA	1677	1.39 ± 0.34	0.035	0.383		0.029	0.425		0.06	0.162
					(-0.044-0.144)			(-0.042-0.100)			(-0.024-0.145)	
Girls												
	LDL-C	AA	46	2.26 ± 0.69								
		GG+GA	1650	2.580.80	0.317	0.008		0.319	0.008		0.314	0.011
					(0.082-0.552)			(0.084-0.554)			(0.071-0.557)	
	TC	AA	46	3.77 ± 0.74								
		GG+GA	1651	4.13 ± 0.87	0.365	0.005		0.36	0.005		0.366	0.006
					(0.111-0.618)			(0.107-0.614)			(0.105-0.627)	
	TG^a^	GG+GA	1650	0.94 ± 0.43								
		AA	46	0.75 ± 0.29	0.216	0.001		0.223	< 0.001		0.224	0.001
					(0.091-0.340)			(0.102-0.344)			(0.098-0.349)	
	HDL-C	AA	46	1.46 ± 0.31								
		GG+GA	1651	1.42 ± 0.30	-0.036	0.424		-0.045	0.270		-0.044	0.304
					(-0.125-0.052)			(-0.126-0.035)			(-0.129-0.040)	

Traits are expressed as the mean ± standard deviation.

Model 1: Adjusted for age. Model 2: Adjusted for model 1 + BMI. Model 3: Adjusted for age model 1 + obesity statues.

TC, total cholesterol; TG, triglyceride; HDL-C, high-density lipoprotein cholesterol; LDL-C, low-density lipoprotein cholesterol; CI, confidence interval.

a natural logarithm-transformed.

## Discussion

In this study, we examined the SNP rs6499640 in *FTO* in a Chinese children population. Our results indicated that the association of this SNP with lipid levels is independent of obesity. This supports the suggested activity of FTO protein, in conjunction with C/EBP, as a co-activator of PPAR gamma, which is involved in adipocyte functions, such as lipid metabolism and differentiation (Wu Q *et al.*, 2010).

The association between *FTO* and lipid levels is difficult to clarify. Many factors can affect the authenticity of the association. The reason for the discrepant results among Chinese studies might be due to the different ages of the samples. We recruited children aged 6-18 years and surveyed the effects of rs6499640 on lipid levels. However, a study in Chinese adolescents (n = 842) aged from 14 to 18 years did not show associations between rs6499640 and TC and TG (Wu *et al.*, 2014). The interference of environmental effects on genetics factors is lower in young people than in older people and thus, young populations are the ideal group to study the effects of genetic factors on lipids metabolism. The non-significant association between rs6499640 and dyslipidemia in our study might be due to a lack of case samples. With time, there are accumulative risk effects on dyslipidemia, but we studied children and adolescents and found results consistent with several previous studies (Fang *et al.*, 2010; Muñoz-Yáñez *et al.*, 2016).

This study also suggested sex-dependent effects of SNP rs6499640 on lipid levels. The association could be partly explained by the fact that boys and girls differ in fat mass percentage and adipocytokines. Our previous studies have found differences in leptin and adiponectin levels between male and female students, and these adipocytokines are related to insulin resistance, while fasting insulin and insulin sensitivity might play important roles in the achievement of critical lipid levels; (Sinaiko *et al.*, 2001; Recasens *et al.*, 2004; Mi *et al.*, 2010). Another reason might be the interaction between genes and environment in males and females. In this study, boys and girls were in different puberty stages (Table S3), which have large behavioral, physiological, and endocrine changes (Wang *et al.*, 2012). Further investigations are needed to understand this mechanism in detail.

Several studies have described the associations between *FTO* and lipid levels. One study investigated the association between the *FTO* SNP rs9939609, *PPARG2* SNP rs1801282, *ADIPOQ* SNPs rs4632532, and rs182052 with obesity-related traits in Mexican children with a high prevalence of obesity (43.7%) (Muñoz-Yáñez *et al.*, 2016). They found that the variant A of SNP rs9939609 was associated with LDL-C after adjusting for age, gender, and BMI. Furthermore, Qureshi *et al.* (2017) suggested that the rs3751812-T allele is a risk factor that may increase LDL-C and decrease HDL-C in a study of 475 Pakistani adults. Another study investigated the associations between SNP rs9939609 and metabolic parameters in boys and girls separately (Elouej *et al.*, 2016). They found that the *FTO* rs9939609 showed a significant association with TC among Tunisian women. When BMI was further adjusted, the association of this polymorphism with TC remained significant. Fang *et al*. (2010) found that *FTO* rs9939609 was associated with log TG in Chinese children, and Kring *et al.* (2008) observed an association between *FTO* rs9939609 and low HDL-C, but the associations disappeared after adjusting for BMI. Many studies have adjusted the effects of *FTO* SNP on lipid levels for BMI, and most found positive results, as did this study. From the studies that analyzed this association, we can observe that the minor allele frequency (MAF) of the *FTO* SNPs included in those studies is higher than that of the respective populations (Table S4). That may be one of the reasons those articles have positive results; the MAF of different *FTO* variants showed that this gene may play an important part in the common forms of lipid levels. We also investigated the linkage disequilibrium about the *FTO* SNPs in CHB populations (https://www.ncbi.nlm.nih.gov/variation/tools/1000genomes/, https://www.broadinstitute.org/haploview/haploview; the results (rs6499640 vs rs3751812: D’=1, r^2^=0.001, rs6499640 vs rs9939609: D’=1, r^2^=0.004) provided direct evidence that the rs6499640 SNP influences lipid levels.

Our study shows that the associations of *FTO* with lipid levels remained significant after further adjustment for BMI, suggesting that the effects of *FTO* on obesity and lipid levels are independent of each other, but we didn’t find the associations of interactions between *FTO* and obesity status with lipid levels (Table S5). Further study is needed to clarify whether obesity changes the lipid profile due to disturbed metabolism or the abnormal lipid profile leads to obesity.

In conclusion, we demonstrated for the first time that the SNP rs6499640 in *FTO* is associated with TC, LDL-C, and TG in Chinese girls. No significant associations were found in boys. These novel findings identify a new risk locus associated with lipid levels in children. The function of *FTO* remains to be further studied to help elucidate the pathogenic role of *FTO* in lipid levels.

## Supplementary material

The following online material is available for this article:

Table S1 - Body mass index reference for screening overweight and obesity in Chinese children and adolescents (kg/m^2^).

Table S2 - The effect of FTO on lipid levels.

Table S3 - Tanner stage in boys and girls.

Table S4 - MAF of studies on the association between FTO and lipid levels.

Table S5 - Interactions between rs6499640 and obesity status on lipid levels.
