# Supplementary material for: Gender-specific association of the rs6499640 polymorphism in the FTO gene with plasma lipid levels in Chinese children
Source: Genet Mol Biol. 2018 Jun 4;41(2):397–402. doi: 10.1590/1678-4685-GMB-2017-0107 (PMC6082231; doi:10.1590/1678-4685-GMB-2017-0107)
Supplement: Supplementary file 1 [file 1415-4757-GMB-1678-4685-GMB-2017-0107-s001.pdf]

Supplementary Material to "Gender-specific association of the rs6499640 polymorphism in the *FTO* gene with plasma lipid levels in Chinese children"

Table S1 - Body mass index reference for screening overweight and obesity in Chinese children and adolescents (kg/m^2).

Age (years)	Boys		Girls	
	Overweight	Obese	Overweight	Obese
6	16.8	18.4	16.7	18.4
7	17.4	19.2	17.2	18.9
8	18.1	20.3	18.1	19.9
9	18.9	21.4	19.0	21.0
10	19.6	22.5	20.0	22.1
11	20.3	23.6	21.1	23.3
12	21.0	24.7	21.9	24.5
13	21.9	25.7	22.6	25.6
14	22.6	26.4	23.0	26.3
15	23.1	26.9	23.4	26.9
16	23.5	27.4	23.7	27.4
17	23.8	27.8	23.8	27.7
18	24.0	28.0	24.0	28.0

Ji CY and Working Group on Obesity in China (2005) Biomed Environ Sci 18:390-400.
