# Supplementary material for: Gender-specific association of the rs6499640 polymorphism in the FTO gene with plasma lipid levels in Chinese children
Source: Genet Mol Biol. 2018 Jun 4;41(2):397–402. doi: 10.1590/1678-4685-GMB-2017-0107 (PMC6082231; doi:10.1590/1678-4685-GMB-2017-0107)
Supplement: Supplementary file 2 [file 1415-4757-GMB-1678-4685-GMB-2017-0107-s002.pdf]

Supplementary Material to "Gender-specific association of the rs6499640 polymorphism in the *FTO* gene with plasma lipid levels in Chinese children"

Table S2 - Associations of rs6499640 with high LDL-C, high TC, high TG and low HDL-C in children under dominant model

Gender	Phenotype	Model 1				Model 2				Model 3			
		OR	95%CI	*p*	Power	OR	95%CI	*p*	Power	OR	95%CI	*p*	Power
Total	high LDL-C	1.773	0.856-3.669	0.123	0.999	1.792	0.865-3.712	0.116	0.999	1.797	0.868-3.723	0.115	0.999
	high TG	1.371	0.661-2.844	0.396	0.999	1.43	0.683-2.993	0.343	0.999	1.456	0.697-3.04	0.318	0.999
	high TC	1.714	0.746-3.938	0.204	0.993	1.7	0.740-3.908	0.212	0.992	1.692	0.736-3.890	0.216	0.991
	low HDL-C	1.348	0.672-2.703	0.401	0.774	1.466	0.718-2.993	0.293	0.937	1.511	0.742-3.079	0.256	0.965
	dyslipidemia	1.480	0.927-2.361	0.101	0.999	1.555	0.965-2.506	0.070	0.999	1.568	0.975-2.522	0.064	0.999
		Model 4				Model 5				Model 6			
Boys	high LDL-C	1.055	0.474-2.347	0.895	0.063	1.066	0.478-2.375	0.877	0.069	1.071	0.480-2.387	0.868	0.072
	high TG	0.794	0.372-1.695	0.551	0.279	0.827	0.378-1.806	0.633	0.206	0.846	0.388-1.842	0.673	0.171
	high TC	1.443	0.517-4.033	0.484	0.552	1.442	0.516-4.030	0.485	0.550	1.443	0.516-4.031	0.484	0.552

Gender	Phenotype	Model 1				Model 2				Model 3			
		OR	95%CI	*p*	Power	OR	95%CI	*p*	Power	OR	95%CI	*p*	Power
	low HDL-C	1.551	0.604-3.984	0.362	0.854	1.783	0.676-4.701	0.243	0.979	1.822	0.690-4.811	0.226	0.986
	dyslipidemia	1.120	0.634-1.980	0.696	0.180	1.174	0.651-2.116	0.594	0.314	1.185	0.658-2.131	0.572	0.345
	high LDL-C	6.849	0.939-49.960	0.058	0.999	6.916	0.948-50.465	0.056	0.999	6.832	0.936-49.859	0.058	0.999
	high TG	1.59×10^8	-	0.997	0.999	1.61×10^8	-	0.997	0.999	1.64×10^8	-	0.997	0.999
Girls	high TC	2.269	0.544-9.454	0.261	0.998	2.215	0.531-9.248	0.275	0.998	2.175	0.520-9.096	0.287	0.996
	low HDL-C	1.125	0.398-3.180	0.824	0.107	1.149	0.400-3.299	0.796	0.130	1.222	0.424-3.521	0.71	0.222
	dyslipidemia	2.457	1.034-5.837	0.042 [a]	0.999	2.566	1.073-6.135	0.034 [a]	0.999	2.591	1.085-6.190	0.032 [a]	0.999

Model 1: Adjusted for age and gender. Model 2: Adjusted for model 1 + BMI. Model 3: Adjusted for model 1 + obesity statues. Model 4: Adjusted for age. Model 5: Adjusted for model 4 + BMI. Model 6: Adjusted for model 4 + obesity statues.

TC, total cholesterol; LDL-C, low-density lipoprotein cholesterol; HDL-C, high-density lipoprotein cholesterol; TG, triglycerides; OR, odds ratio; CI, confidence interval.

[a] *P*-value has nonsignificant after FDR test is applied.
