# Supplementary material for: Gender-specific association of the rs6499640 polymorphism in the FTO gene with plasma lipid levels in Chinese children
Source: Genet Mol Biol. 2018 Jun 4;41(2):397–402. doi: 10.1590/1678-4685-GMB-2017-0107 (PMC6082231; doi:10.1590/1678-4685-GMB-2017-0107)
Supplement: Supplementary file 3 [file 1415-4757-GMB-1678-4685-GMB-2017-0107-s003.pdf]

Supplementary Material to "Gender-specific association of the rs6499640 polymorphism in the *FTO* gene with plasma lipid levels in Chinese children"

Table S3 - Tanner stage in boys and girls.

Tanner stage	Boys	Girls
I(%)	664(37.3)	336(19.5)
II(%)	270(15.2)	218(12.7)
III(%)	259(14.5)	209(12.1)
IV(%)	204(11.4)	611(35.5)
V(%)	303(17.8)	323(18.8)

The Tanner stages were measured using male testicular volume and female breast Tanner staging (Marshall *et al*., 1969, 1970). There were 81 and 25 missing data in boys and girls respectively.

References

Marshall WA and Tanner JM (1969) Variations in pattern of pubertal changes in girls. Arch Dis Child 44:291-303.

Marshall WA and Tanner JM (1970) Variations in the pattern of pubertal changes in boys. Arch Dis Child 45:13-23.
