# Supplementary material for: Gender-specific association of the rs6499640 polymorphism in the FTO gene with plasma lipid levels in Chinese children
Source: Genet Mol Biol. 2018 Jun 4;41(2):397–402. doi: 10.1590/1678-4685-GMB-2017-0107 (PMC6082231; doi:10.1590/1678-4685-GMB-2017-0107)
Supplement: Supplementary file 4 [file 1415-4757-GMB-1678-4685-GMB-2017-0107-s004.pdf]

Supplementary Material to "Gender-specific association of the rs6499640 polymorphism in the *FTO* gene with plasma lipid levels in Chinese children"

Table S4 - MAF of studies on the association between FTO and lipid levels.

Reference	Genotype	Region/ethnicity	MAF
Kring *et al.* (2008)	rs9939609-A	Japaness	0.464
Fang *et al*. (2010)	rs9939609-A	Chinese	0.122
Muñoz-Yáñez *et al*. (2016)	rs9939609-A	Mexican	0.350
Qureshi *et al*. (2016)	rs3751812-T	Pakistani	0.312
Elouej *et al*. (2016)	rs9939609-A	Tunisian	0.374
present study	rs6499640-G	Chinese	0.167

MAF, minor allele frequency.

References

Elouej S, Belfki-Benali H, Nagara M, Lasram K, Attaoua R, Sallem OK, Kamoun I, Chargui M, Romdhane L, Jamoussi H, *et al.* (2016) Association of rs9939609 Polymorphism with Metabolic Parameters and FTO Risk Haplotype Among Tunisian Metabolic Syndrome. Metab Syndr Relat 14:121-128.

Fang H, Li Y, Du S, Hu X, Zhang Q, Liu A and Ma G (2010) Variant rs9939609 in the FTO gene is associated with body mass index among Chinese children. BMC Med Genet 11:136.

Kring SI, Holst C, Zimmermann E, Jess T, Berentzen T, Toubro S, Hansen T, Astrup A, Pedersen O and Sørensen TI (2008) FTO gene associated fatness in relation to body fat distribution and metabolic traits throughout a broad range of fatness. PLoS One 3:e2958.

Muñoz-Yáñez C, Pérez-Morales R, Moreno-Macías H, Calleros-Rincón E, Ballesteros G, González RA and Espinosa J (2016) Polymorphisms FTO rs9939609, PPARG rs1801282 and ADIPOQ rs4632532 and rs182052 but not lifestyle are associated with obesity related-traits in Mexican children. Genet Mol Biol 39:547-553.

Qureshi SA, Mumtaz A, Shahid SU and Shabana NA (2017) Rs3751812, a common variant in fat mass and obesity-associated (FTO) gene, is associated with serum high- and low-density lipoprotein cholesterol in Pakistani individuals. Nutrition 39-40:92-95.
