# Supplementary material for: Gender-specific association of the rs6499640 polymorphism in the FTO gene with plasma lipid levels in Chinese children
Source: Genet Mol Biol. 2018 Jun 4;41(2):397–402. doi: 10.1590/1678-4685-GMB-2017-0107 (PMC6082231; doi:10.1590/1678-4685-GMB-2017-0107)
Supplement: Supplementary file 5 [file 1415-4757-GMB-1678-4685-GMB-2017-0107-s005.pdf]

Supplementary Material to "Gender-specific association of the rs6499640 polymorphism in the *FTO* gene with plasma lipid levels in Chinese children"

Table S5 - Interactions between rs6499640 and obesity status on lipid levels.

			Total		Boys		Girls	
			AA	GG+GA	AA	GG+GA	AA	GG+GA
			p	*p*	*p*	*p*	*p*	*p*
high TG	obesity	Yes	0.224	0.028	0.492	0.327	0.999	0.998
		No		0.566		0.336		0.998
high TC	obesity	Yes	0.699	0.707	0.972	0.639	0.999	0.935
		No		0.315		0.575		0.403
high LDL-C	obesity	Yes	0.554	0.071	0.686	0.470	0.999	0.096
		No		0.209		0.975		0.132
low HDL-C	obesity	Yes	0.139	0.010	0.232	0.058	0.329	0.084
		No		0.601		0.479		0.930

high TC: TC ≥5.18 mmol/L; high TG: TG ≥1.70 mmol/L; high LDL-C: LDL-C ≥3.37 mmol/L; low HDL-C: HDL-C ≤1.04 mmol/L (Editorial Board of Chinese Journal of Pediatrics *et al*., 2009, Chin J Pediatr 47:426-428).

After FDR test, no significant association was found.
